# Investigation of antibacterial and anticancer activities of copper, aluminum and nickel doped zinc sulfide nanoparticles

**DOI:** 10.1038/s41598-024-68631-0

**Published:** 2024-08-20

**Authors:** Tariq Munir, Arslan Mahmood, Irfan Ali, Numan Abbas, Amjad Sohail, Arshia, Yasin Khan

**Affiliations:** 1 Department of Physics, Government College University Faisalabad (GCUF), Allama Iqbal Road, Faisalabad, 38000 Pakistan; 2 College of Physics and Information Technology, Shaanxi Normal University, 710119 Xian, Shaanxi People’s Republic of China; 3 Department of Electrical Engineering, King Saud University, Riyadh, Saudi Arabia

**Keywords:** Zinc sulfide NPs, Biological potential, Cell viability, Antibacterial, Hyperthermia, Biophysics, Nanoscience and technology

## Abstract

First time compared the different metals doped ZnS nanoparticles for antibacterial and liver cancer cell line. In this study, copper, aluminum and nickel doped ZnS NPs were synthesized via co-precipitation method. The XRD analysis was confirmed the presence of cubic crystal structure and crystallite size decreased from 6 to 3 nm with doping elements. While as SEM micro-grains were revealed slightly irregular and agglomerated morphology with the presence of dopant elements. The presence of different dopant elements such as Cu, Al and Ni in ZnS NPs was identified via EDX analysis. The FTIR results demonstrate various vibrational stretching and bending modes attached to the surface of ZnS nanomaterials. After that the well diffusion method was used to conduct in-vitro bioassays for evaluation of antibacterial and anticancer activities against *E.coli* and *B.cereus*, as well as HepG2 liver cancer cell line. Our findings unveil exceptional results with maximum inhibition zone of approximately 9 to 23 mm observed against *E.coli* and 12 to 27 mm against B*.cereus*, respectively. In addition, the significant reduction in cell viability was achieved against the HepG2 liver cancer cell line. These favorable results highlight the potential of Ni doped ZnS NPs for various biomedical applications. In future, the doped ZnS nanomaterials will be suitable for hyperthermia therapy and wound healing process.

## Introduction

According to recent report the cancer is second leading disease in worldwide. Likewise, lung cancer disease is prominent in males, while breast carcinoma affects females significantly. These malignancies arise from a multitude of etiological factors, encompassing tobacco, dietary habits, radiation exposure, hormonal imbalances and presence of pathogenic microorganisms in environment cause different diseases^1,2^. Despite the availability of diverse cancer therapies such as radio and chemotherapy and their administration often accompanies a range of adverse effects. These include cardio cytotoxicity (perturbation of cardiac cells), nephrotoxicity (toxicity targeting renal function), myelosuppression (suppression of hematopoiesis), neurotoxicity (neuronal damage), hepatotoxicity (hepatic impairment), gastrointestinal toxicity (detrimental effects on the gastrointestinal tract), mucositis (mucous membrane inflammation), and alopecia (hair loss)^3–8^. Furthermore, another one of the most common diseases affecting individual is caused by bacterial pathogens, leading to the manifestation of symptoms such as diarrhea and vomiting. These symptoms are primarily attributed to the presence of specific bacterial species, including *E.coli, Campylobacter spp*., *Salmonella spp*., *Shigella spp*., and *Vibrio spp*. These bacteria are recognized as significant causative agent of gastrointestinal infections and contributing to the prevalence of diarrhea and vomiting, which pose substantial health challenges on a global scale^9–13^. Antibiotics are used extensively but they have several side effects like diarrhea, nausea, vomiting including fungal infections and allergic reactions^14–16^. Therefore, drugs are replaced by nanotechnology because of their unique physical, chemical and biological properties, which is the novel and unharmed technique for cancer therapeutic applications and antibacterial infection^17–20^.

In addition, graphene base nanocomposite and functionalized graphene oxide nanocomposite (NCs) also provided the significant antimicrobial and anticancer results against different strains of bacteria and fungi and multiple type of cell line. Maruthupandy et al.^40^ reported that the photocatalytic degradation of dye was improved with the help of graphene ZnO NCs. The ferrite base nanocomposites play the central role for the treatment of cancer via hyperthermia therapy and drugs delivery. While as considerable research efforts were devoted to the synthesis of nanocomposites due to their favorable biocompatibility, non-toxicity and smooth flow inside blood^21^. Likewise, silver NPs derived from the extract of *Origanum Vulgare* plant leaves, have exhibited notable efficacy in anticancer treatments against human liver cancer cell line (HepG2) and antibacterial activity against various bacterial strains, including drug-resistant ones^22^. After that the silver doped zinc oxide NPs coated with polyethylene glycol, have demonstrated anticancer effects on UVB-induced skin cancer cells. It also exhibited antibacterial activity against opportunistic skin pathogens^23^. The ZnS NPs were synthesized and found to inhibit the growth of MCF-7 cancer cells, while exhibiting lower toxicity compared to *Stevia* extract alone^24^. Additionally, ZnS NPs synthesized using a co-precipitation method with specific additives demonstrated potent antibacterial properties^25^. Moreover, ZnS NPs synthesized via sonochemical method exhibited inhibitory effects on cell migration and invasion in breast cancer cells and promoted wound healing in MCF-7 cell^26^.

In current study was focused on the synthesis of pure and doped ZnS NPs by using co-precipitation method for the evaluation of anticancer and antibacterial applications. The synthesized NPs were subjected to comprehensive characterization employing techniques including XRD, SEM, EDX, FTIR and the well diffusion method for antibacterial and anticancer activities assessment. The results revealed that Cu doped ZnS NPs exhibit enhanced antibacterial and anticancer properties displaying a significant level of effectiveness. In addition Table 1 shows the comparison of oxide and sulfide base nanomaterials for different applications.

**Table 1:** Shows the various parameters of metal oxide and sulfide base nanomaterials.

Nanomaterials	Synthesis method	Size of NPs	Applications	References
ZnO/CuO NCs	Green synthesis method	7.5 to 8.12 nm	Biodegradation	^27^
			Cytocompatibility	
			Wound healing	
ZnO/CuO NCs	Green synthesis process	10 nm	Bactericidal	^28^
			Cytocompatibility	
			Wound healing	
ZnO–CuO NCs	Green synthesis process	7.5 to 8.12 nm	Antimicrobial properties	^29^
ZnO/C/Ca–NCs	Green synthesis process	–	Antimicrobial properties	^30^
CuO–NPs	Green synthesis process	–	Bactericidal	^31^
			Cytocompatibility	
Ni dopant ZnO-NPs	Co-precipitation method	19 to 21 nm	Biomedical applications	^32^
ZnO-NPs	Sol–gel technique	20 nm	Hematological and serological profile of *Catla catla* fish	^33^
Mn-doped ZnS-NPs	Chemical synthesis process	4 to 7 nm	Electro-optic applications	^34^
Cu doped and starch capped ZnS nanocrystals	Microwave irradiation	3.48 nm	Photoluminescence	^35^
Eu-doped ZnS-NPs	Sol–gel wet chemical method	7.6 to 8.5 nm	Photoluminescence	^36^
Zn and Ni doped cobalt ferrite	sol–gel method	7.2 to 5.1 nm crystallite size	Antibacterial	^37^
Cr doped ZnO NPs	Ultrasonic assisted co precipitation method	32.15 to 22.15 nm crystallite size	Antimicrobial	^38^
Pb doped ZnO NPs	Ultrasonic-assisted co-precipitation method	34 to 20 nm crystallite size	Antibacterial	^39^
			Photocatalytic degradation	
G-ZnO NCs	Chemical precipitation method	8 to 12 nm	Photocatalytic degradation	^40^
Zinc oxide nanosheets	Biosynthesis process	–	Antibiofilm activity	^41^

## Experiment

### Precursors

The high grade precursors such as zinc acetate (98%), sodium sulfate (99%), sodium hydroxide (97%), nickel acetate tetrahydrate (99.995%), aluminum acetate dibasic and copper acetate (98%) were purchase from Sigma Aldrich used for the preparation of doped zinc sulfide nanoparticles. The deionized water was purchase from local market. These nanoparticles were employed for their anticancer and antibacterial properties.

### Synthesis of ZnS NPs

The ZnS NPs were synthesized with the help of sodium sulfide (Na_2_S, 0.03 M) with zinc acetate (Zn (CH_3_CO_2_)_2_, 0.02 M) under continuous stirring on a magnetic stirrer at temperature 90 °C for 30 min. The sodium hydroxide was used to adjust the pH upto 13 (NaOH, 0.07 M). The resulting NPs were subsequently separated from the reaction mixture using filtration and then subjected to a drying process in an oven set at 140 °C for 4 h. The dried ZnS NPs were further processed by grinding them using a mortar and pestle.

### Copper, aluminum and nickel doped ZnS NPs

The direct addition method was employed to synthesis of ZnS NPs with proper Al as doping element. In this method, a solution of aluminum acetate (0.03 M) was added directly into ZnS solution by increasing temperature 100 °C for 40 min. This ensured that the successful incorporation of Al into ZnS materials. The subsequent steps of the synthesis are followed the same procedure as described previously, including stirring, pH adjustment, filtration, drying in an oven at 140 °C and grinding of the Al doped ZnS nanoparticles. The same process was used for copper and nickel doping respectively.

### Characterization of doped ZnS NPs

**Figure1:**
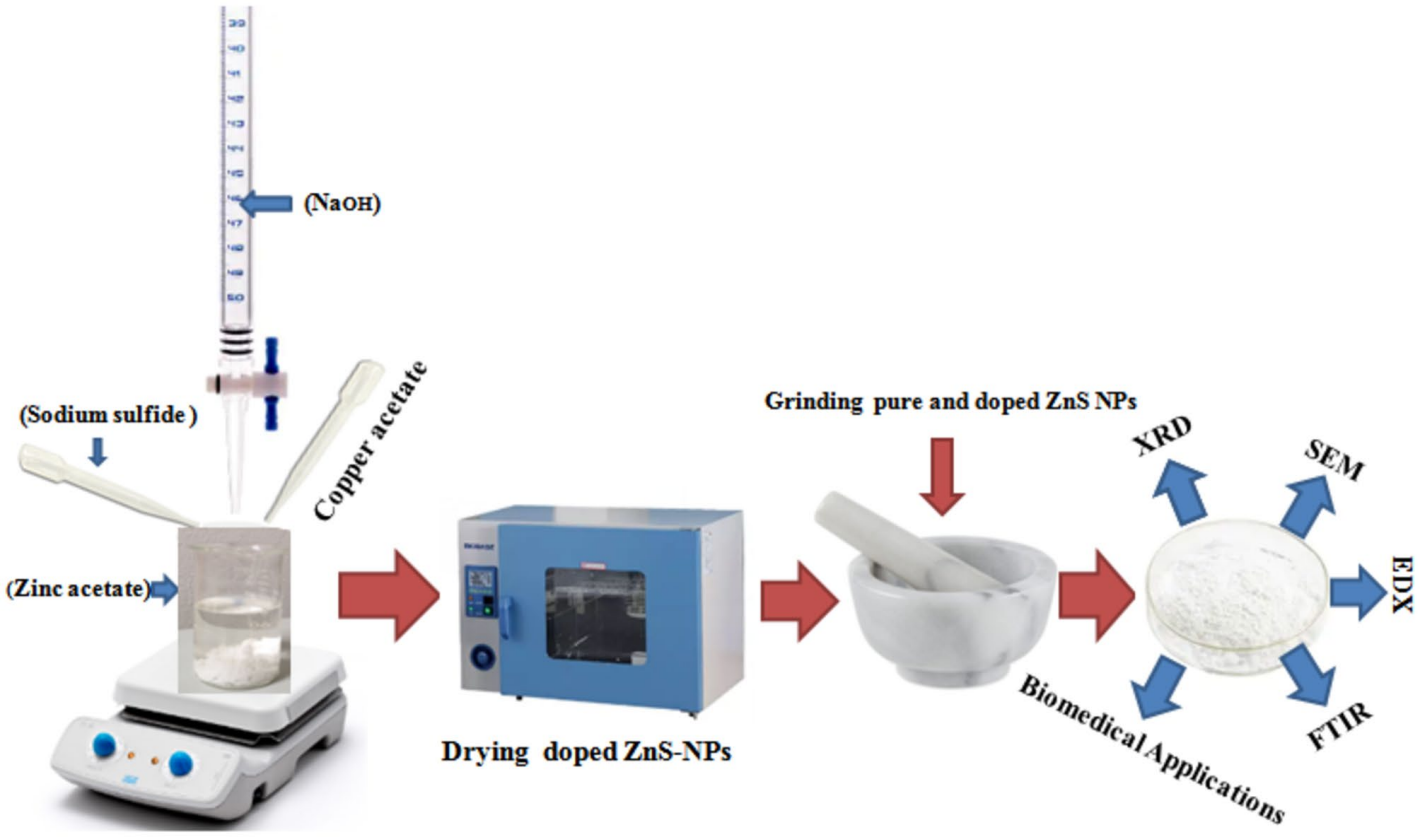
Synthesis methodology of doped ZnS NPs.

Multiple characterization techniques were used to analyze the doped ZnS NPs. The XRD analysis was utilized with Cu-Kα radiation to investigate the structural information (D8 Advance and X’Pert3 MRD XL instruments). Nova Nano (450) SEM was used to observe the surface morphology of micro grains was appeared in SEM micrograph images. The presence of elemental composition in doped ZnS NPs was examined via EDX analysis. While as FTIR Bruker spectrometer was used to identify the different functional groups attached with spectrum of undoped and doped ZnS NPs. The Fig. 1 represents the systematic diagram of synthesis methodology of undoped and doped ZnS NPs.

### Bioassay

#### Antibacterial assay

The previous reported bacterial culture process was followed^42^.The antibacterial activity was tested against *E.coli* (ATCC 25922) gram negative and *B.cereus* (ATCC 14579) gram-positive by using pure; Ni, Al and Cu doped ZnS NPs. The antibacterial activity was examined by 40 mg/mL of pure; Co, Al and Ni doped ZnS NPs. The inhibition zone was observed after 24 h at 37 °C.

#### MTT assay of HepG2 cell line

The liver cancer cell line was cultured via 96 well plates and tissue culture flask (25 cm) was used, after that the contribute Hank salts 10% fetal bovine serum in DMEM. Furthermore, after some nonessential amino acids and glutamine 2 mM/L are incubated 24 h at 37 °C. The various concentrations (0, 10, 20, 30 and 40 mg/mL) of pure, Cu, Ni and Al doped ZnS NPs solution incorporated in cultured cells. The same MTT assay process followed as published earlier^43^.

#### Statistical analysis

Data was analyzed by using two-way ANOVA and mean values of different treatments were compared by using least significant difference (LSD) at 95% confidence (P $\le$ 0.05) index with the help of Co-Stat software.

## Results and discussion

### Structural analysis

**Figure 2:**
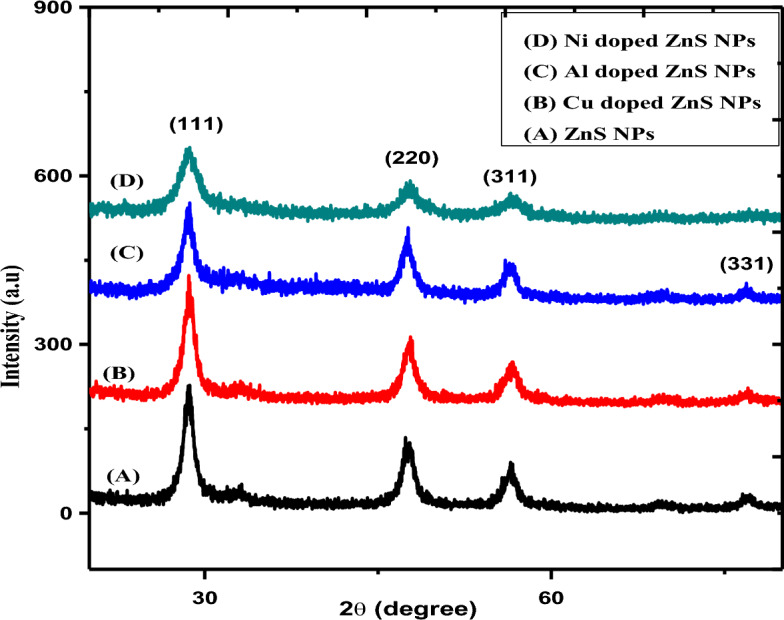
XRD spectrum of Cu, Al and Ni doped ZnS NPs.

Figure 2 presents the XRD spectrum of undoped and doped ZnS NPs with diverse doping elements (Cu, Al and Ni). The XRD spectrum represents distinct diffraction peaks was attributed at various miller indices notably (111), (220), (311) and (331) corresponding different values of 2θ such as 28.5°, 47.8°, 56.4° and 76.9°. While as these indices represents cubic crystal structure was appeared at most prominent peak of undoped and doped ZnS NPs. Furthermore, a comparative analysis between ZnS NPs and Cu, Al and Ni doped ZnS-NPs. The observed diffraction peaks closely match the anticipated diffraction pattern for cubic ZnS ICDD powder diffraction file No. 80–0020^44,45^.There was slightly intensity decreased with Cu, Al and Ni doping agents, but the Ni incorporation into the ZnS lattice becomes evident through a discernible shift in the diffraction peak associated with the (111) plane. The corresponding 2θ value exhibits an increase from 28.86 to 29.12, owing to the smaller ionic radius of Ni (0.83 Å) compared to Zn (0.88 Å). The absence of discernible diffraction peaks corresponding to Ni, NiS, or other impurity phases validates the successful substitution of Ni as the dopant within the ZnS lattice^46^. In addition the sharpness of the peaks decrease with the metal doping agent like Ni, it means that with the Ni doping the crystallinity of ZnS NPs decreased upto significant values. In order to examine the imperfections occurring during crystal growth, several parameters including crystallite size and peak width were computed. These calculations were undertaken to gain insights into the quality of the crystal and to evaluate the presence of any defects or distortions within its structure^47^.The Scherrer’s Eq. (1) was preferred to examine the crystallite size of undoped, Cu, Al and Ni doped ZnS NPs. It was also investigated that the crystallite size of ZnS NPs were decreased with doping agents are expressed in (Table 2).

1 $$D = \frac{k\lambda }{{\beta cos\theta }}$$

**Table 2:** FWHM and crystallite size of doped ZnS NPs.

Nanomaterials	FWHM (degree)	Crystallite size (nm)
ZnS NPs	1.34204	6
Cu doped ZnS NPs	1.37281	5.8
Al doped ZnS NPs	1.73979	4.6
Ni doped ZnS NPs	2.5067	3.3

### Surface analysis

**Figure 3:**
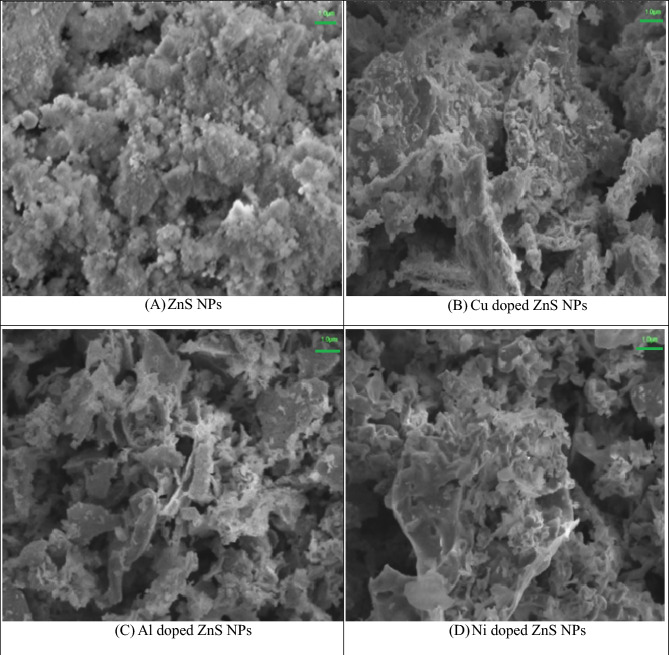
SEM micrographs of metals doped ZnS NPs.

**Figure 4:**
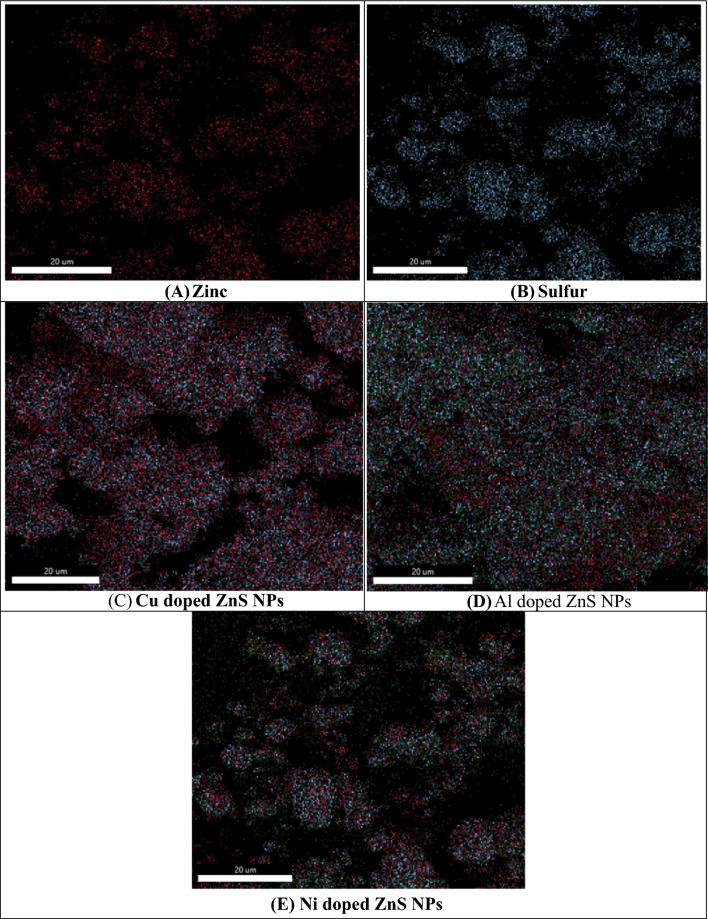
SEM images for the identification of nanocomposite.

The surface morphology of synthesized ZnS NPs and various elements (Cu, Al and Ni) doped ZnS NPs were investigated with SEM analysis. The Fig. 3 exhibits the surface morphology of pure ZnS, Cu, Al and Ni doped ZnS NPs. The entire micrographs demonstrate that ZnS possess irregular and non-uniform grains with slight agglomeration^48^. It was also observed that the agglomeration increased with Cu doping elements in ZnS NPs. Additionally, increase the agglomeration rate which provide evidence of the effective dispersion of Al, Ni and Cu on the surface of ZnS NPs. All the micrograph was collected 1 µm magnification level and these graph shows that flake like surface morphology was observed after doping Al, Ni and Cu in ZnS NPs. Dawngliana et al.^49^ provided the information about the micrographs of ZnS NPs and also reported that nearly spherical particles was observed by calcination temperature upto 900 °C and doping of Sm^+3^ ion and similar morphology was observed in case of nanocomposites^49^. Figure 4 represents the identification of different elements in SEM micrographs. All the collected micrographs from (A to E) represent the different colors of Zn and sulfur and other metals doped ZnS base nanocomposite. While as all colored images were collected same magnification level 20 µm.

### EDX analysis

**Figure 5:**
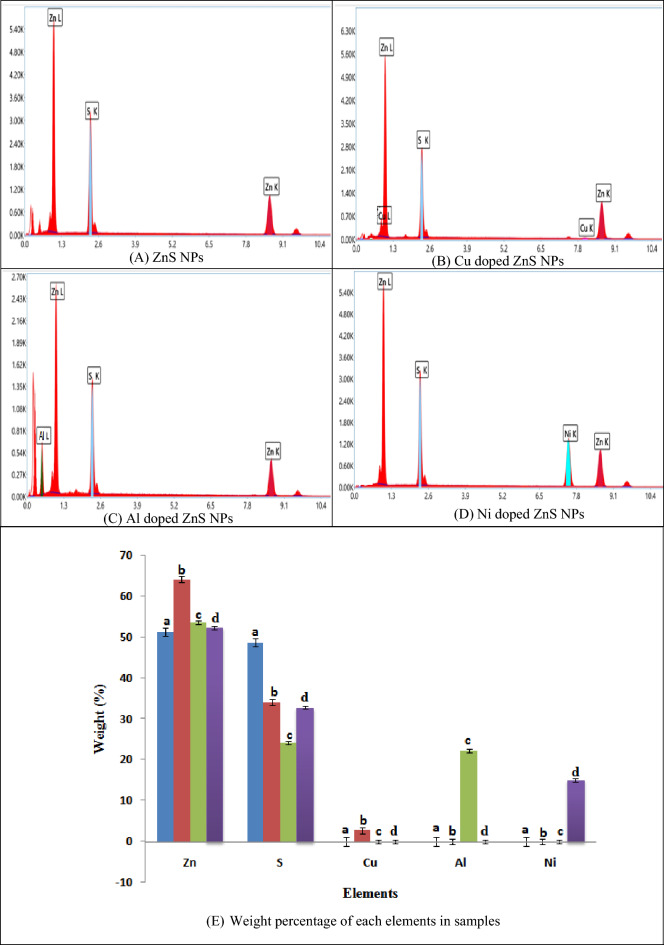
Elemental composition of doped ZnS-NPs.

The elemental composition and purity/clarity related information of each sample was collected by EDX analysis. Figure 5 shows the elemental composition and weight percentage of each element in undoped and doped ZnS NPs samples. Here, (A) ZnS NPs sample represents the Zn and S intense spectra, (B) Cu doped ZnS NPs express Zn, S and Cu intense peaks, (C) Al doped ZnS NPs indicate the intense peaks of Zn, S and Al and (D) Ni doped ZnS NPs shows the peaks of Zn, S and Ni but (D) bar chat of weight percent of each element present in all samples^50,51^. In addition a, b, c and d label on each bar chat indicate the presence of each element in all samples and also represents the weight percent. The “a” represents the percentage of each element and 1st sample and similarly “d” represents the percentage of each element in last sample.

### FTIR analysis

**Figure 6:**
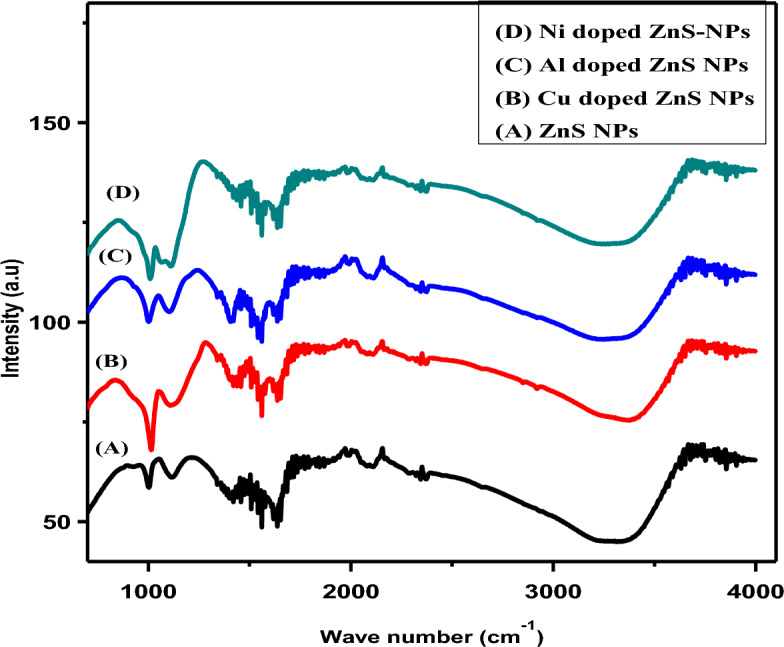
FTIR pattern of doped ZnS NPs.

Figure 6 represents the variation of rotational and vibrational modes attached on the spectrum of undoped, copper, aluminum and nickel doped ZnS NPs was identified with the help of FTIR analysis. The strong band was appeared from 508 to 620 cm^−1^ indicates the existence of ZnS NPs. While as the peak was obtained at 1384 cm^−1^ shows nitrate group^52^. The presence of bending OH (1531 cm^−1^) and at 3350 cm^-1^ shows stretching OH mode. The spectrum shows the band at 1620 cm^−1^ which represents CO_2_ functional groups. The few other bands were observed at 1116 to 976 cm^−1^ which also represents Zn–S mode attached on spectrum. The same bands were reported Selvaraj and his group members in 2022^53^. In addition, Devi B et al.^35^ reported similar study pure and Cu doped ZnS NPs which used starch as coating agent and all the functional groups similar to compare-able with current study. Alwany et al.^53^ provided the information about lead doped ZnS NPs and the FTIR analysis indicated that the wavenumber shift toward shorter wavelength with doping agents^54^. Overall analysis shows that the current wavenumber similar to all previous reported literature of undoped, Cu and Ni doped ZnS NPs and non-availability of Al doped ZnS NPs. The Al doped ZnS NPs was first time reported in current study.

### Anticancer activity

**Figure 7:**
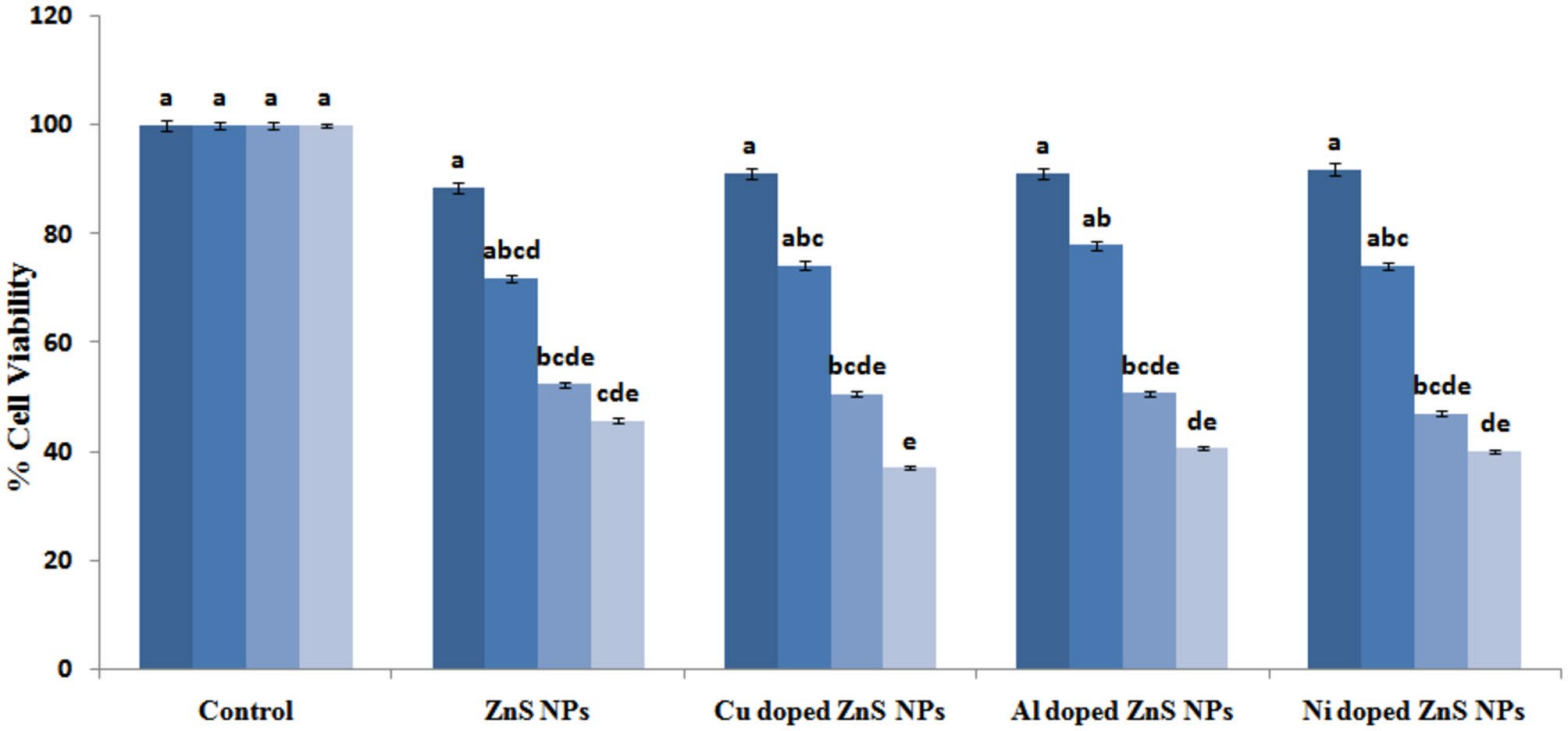
Cell viability of doped ZnS NPs against HepG2 cell line.

**Figure 8:**
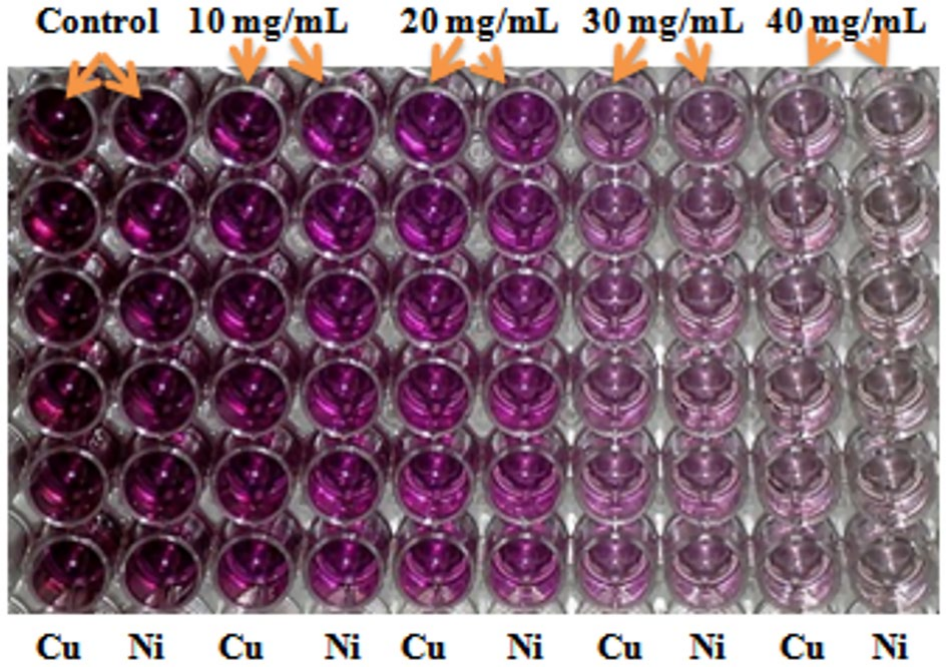
MTT assay of Cu and Ni doped ZnS NPs.

Figure 7 shows that anticancer activity of ZnS nanoparticles both undoped and doped with Cu, Al and Ni elements was evaluated against the HepG2 liver cancer cell line. Remarkably, the results demonstrated that Cu doped ZnS nanoparticles exhibited a substantial decrease in cell viability, approximately at 37 from 100%. Equally but impressive was the enhanced efficiency of Al-doped ZnS nanoparticles, showing a significant reduction in cell viability of approximately 40%. While the cell viability reduction was still considerable for pure ZnS nanoparticles (around 45%) and Ni-doped ZnS nanoparticles (approximately 40%), the remarkable performance of Cu doped ZnS NPs variants highlights their potential as promising candidates for combating liver cancer cell line^55^. The bar chat which represents the value of dose (0, 10, 20, 30 and 40 mg/mL) of nanoparticles solution of undoped and doped ZnS NPs used to kill HepG2 cell line. While as data was analyzed with the help of two-way ANOVA and calculate means value to provide most significant results of Ni doped ZnS NPs having P value (P ≤ 0.05). Wei et al*.*^56^ reported that the various metals doped ZnS NPs against HepG2 cell lines and previous results of ZnS sample was compared against HepG2 cell line and current study. Further, current analysis also shows that the Cu doped ZnS NCs also provided significant results against liver cancer cell line as compared to previous literature^56^. Figure 8 represents the MTT assay of Cu and Ni doped ZnS NPs. The Cu and Ni doped ZnS NPs provided most significant results as compared to pure and Al doped ZnS NPs.

### Antibacterial activity

**Figure 9:**
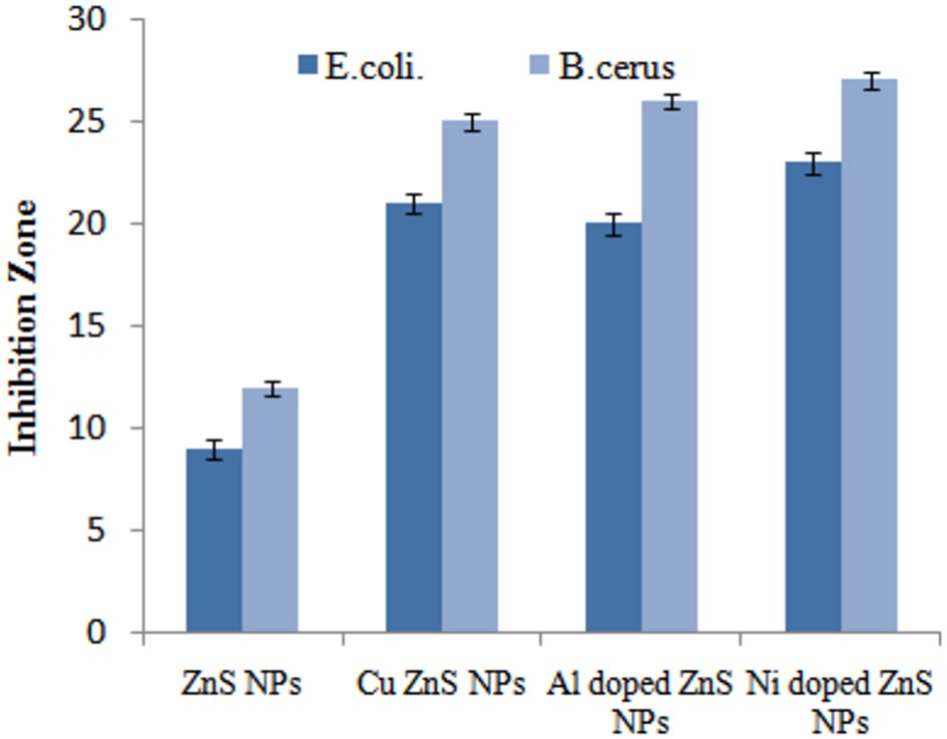
Antibacterial activity of doped ZnS NPs.

**Figure10:**
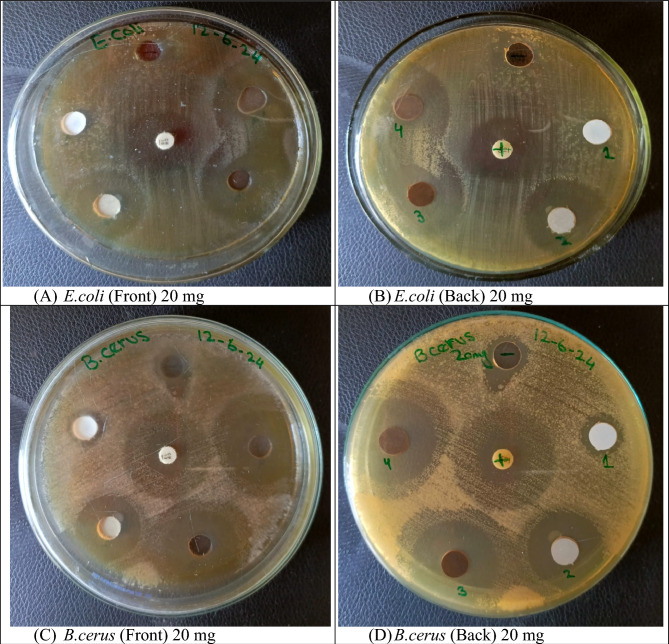
Antibacterial activity of metal doped ZnS NPs.

The Fig. 9 shows antibacterial activity of the synthesized ZnS NPs and (Al, Ni and Cu) doped was assessed against both *E.coli* and *B.cereus* pathogen bacteria using the well diffusion method. The study involved using the same concentration of 20 mg/mL for both ZnS NPs and those doped with Al, Ni, and Cu against the gram-negative/ positive bacterial strains. Pure ZnS NPs exhibited a minor effect on the size of the inhibition zone 9 mm for *E.coli* and 20 mm for *B.cereus* approximately. However, Fig. 9 demonstrated that the size of inhibition zone increased upto significant level by using Cu and Al doped ZnS-NPs. Notably, the maximum inhibition zone of 23 mm against *E.coli* and 27 mm against *B.cereus* was recorded for Ni doped ZnS NPs, indicating their potent antibacterial activity against both bacterial strains. Dhupar et al.^57^ provided the information about indium doped ZnS NPs was used against antibacterial activity by using disc diffusion method. These analysis shows that the inhibition zone increased by increasing indium concentration in ZnS NPs^2,57^. But the current analysis shows that inhibition zone increase by varying doping concentration in ZnS NPs and every element enhance the antibacterial activity upto significant value. Figure 10 represents the inhibition zone of *E.coli* and *B.cereus* was investigated by using pure and doped ZnS NPs. Muthuchamy et al.^58^ reported that the copper and ZnO NPs enhance the antimicrobial activity^58^. While as Rajivgandhi et al.^59^ also provided the information CuO NPs provided less inhibition zone against *P. aeruginosa* and *K. pneumoniae*.

## Conclusion

The co-precipitation method was used for the synthesis of pure, Al, Cu and Ni doped ZnS NPs. The cubic crystal structure was appeared in pure and metals doped ZnS NPs was investigated via XRD analysis. The irregular and non-uniform grains with slight agglomeration doped ZnS NPs was observed with SEM micrograph and the presence of Al, Cu and Ni in ZnS NPs was identify by EDX analysis. After that the different bending and vibrational modes attached on the surface of ZnS NPs spectrum and also identify the presence of doping agents in ZnS spectrum. After material study it was observed that the undoped and doped ZnS nanomaterial was suitable for antibacterial and anticancer activity. It was examined that with the help of well diffusion method the Cu doped ZnS NPs were provided the significant results against *E.coli* and *B.cereus* pathogen bacteria and also calculate the cell viability against liver cancer cell lines. In future the doped ZnS NPs will be used for drugs delivery and remote control process for treatment of cancer.

### Supplementary Information

Supplementary Figures.

## Data Availability

All data generated during this study are included in this published article (Supplementary Information files).

## References

[1] Akram, M. W. *et al.* Chitosan blend iron oxide nanostructure-based biosensor for healthy & malignant tissue glucose/urea detection. *IOP Conf. Ser. Mater. Sci. Eng.* **474**, 012060 (2019). 10.1088/1757-899X/474/1/012060

[2] Munir, T. *et al.* Antimicrobial activities of polyethylene glycol and citric acid coated graphene oxide-NPs synthesized via Hummer’s method. *Arab. J. Chem.* **15**(9), 104075 (2022). 10.1016/j.arabjc.2022.104075

[3] Anjomshoa, M. & Amirheidari, B. Nuclease-like metalloscissors: Biomimetic candidates for cancer and bacterial and viral infections therapy. *Coord. Chem. Rev.* **458**, 214417 (2022). 35153301 10.1016/j.ccr.2022.214417 PMC8816526

[4] Karges, J. Clinical development of metal complexes as photosensitizers for photodynamic therapy of cancer. *AngewandteChem. Int. Ed.* **61**(5), e202112236 (2022). 10.1002/anie.202112236 34748690

[5] Anand, P. *et al.* Cancer is a preventable disease that requires major lifestyle changes. *Pharm. Res.* **25**(9), 2097–2116 (2008). 18626751 10.1007/s11095-008-9661-9 PMC2515569

[6] Zadeh, F. A. *et al.* Autophagy-related chemoradiotherapy sensitivity in non-small cell lung cancer (NSCLC). *Pathol. Res. Pract.* **233**, 153823 (2022). 35398616 10.1016/j.prp.2022.153823

[7] Cowin, P., Rowlands, T. M. & Hatsell, S. J. Cadherins and catenins in breast cancer. *Curr. Opin. Cell Biol.* **17**(5), 499–508 (2005). 16107313 10.1016/j.ceb.2005.08.014

[8] Dapic, V., Carvalho, M. A. & Monteiro, A. N. Breast cancer susceptibility and the DNA damage response. *Cancer Control* **12**(2), 127–136 (2005). 15855896 10.1177/107327480501200210

[9] Sjoling, Å., Sadeghipoorjahromi, L., Novak, D. & Tobias, J. Detection of major diarrheagenic bacterial pathogens by multiplex PCR panels. *Microbiol. Res.* **172**, 34–40 (2015). 25542594 10.1016/j.micres.2014.12.003

[10] Grzybowska-Chlebowczyk, U. *et al.* Clinical course of campylobacter infections in children. *Pediatriapolska* **88**(4), 329–334 (2013).

[11] Xu, C., Akakuru, O. U., Zheng, J. & Wu, A. Applications of iron oxide-based magnetic nanoparticles in the diagnosis and treatment of bacterial infections. *Front. Bioeng. Biotechnol.* **7**, 141 (2019). 31275930 10.3389/fbioe.2019.00141 PMC6591363

[12] Basu, A., Singh, R. & Gupta, S. Bacterial infections in cancer: A bilateral relationship. *Wiley Interdiscip. Rev. Nanomed. Nanobiotechnol.* **14**, e1771 (2022). 34994112 10.1002/wnan.1771

[13] Zhang, Y. J. *et al.* Impacts of gut bacteria on human health and diseases. *Int. J. Mol. Sci.* **16**(4), 7493–7519 (2015). 25849657 10.3390/ijms16047493 PMC4425030

[14] Zhou, L., Chen, F., Hou, Z., Chen, Y. & Luo, X. Injectable self-healing CuS nanoparticle complex hydrogels with antibacterial, anti-cancer, and wound healing properties. *Chem. Eng. J.* **409**, 128224 (2021). 10.1016/j.cej.2020.128224

[15] Bhattacharya, B. & Mukherjee, S. Cancer therapy using antibiotics. *J. Cancer Ther.* **6**(10), 849 (2015). 10.4236/jct.2015.610093

[16] Bayarski, Y. Antibiotics and their types, uses and side effects. Dari: http://hamiltoncountypreppers.org/.Diakses (2006).

[17] Alexis, F. *et al.* New frontiers in nanotechnology for cancer treatment. *Urol. Oncol. Semin. Orig. Investig.* **26**, 74–85 (2008). 10.1016/j.urolonc.2007.03.017 18190835

[18] Thanh, N. T. & Green, L. A. Functionalisation of nanoparticles for biomedical applications. *Nano Today* **5**(3), 213–230 (2010). 10.1016/j.nantod.2010.05.003

[19] Yuan, P., Ding, X., Yang, Y. Y. & Xu, Q. H. Metal nanoparticles for diagnosis and therapy of bacterial infection. *Adv. Healthc. Mater.* **7**(13), 1701392 (2018). 10.1002/adhm.201701392 29582578

[20] Mahmood, A. *et al.* Analyses of structural and electrical properties of aluminium doped ZnO–NPs by experimental and mathematical approaches. *J. King Saud Univ. Sci.* **34**(2), 101796 (2022). 10.1016/j.jksus.2021.101796

[21] Munir, T. *et al.* Treatment of breast cancer with capped magnetic-NPs induced hyperthermia therapy. *J. Mol. Struct.* **1196**, 88–95 (2019). 10.1016/j.molstruc.2019.06.067

[22] Sankar, R. *et al.* Origanumvulgare mediated biosynthesis of silver nanoparticles for its antibacterial and anticancer activity. *Coll. Surf. B Biointerfaces* **108**, 80–84 (2013). 10.1016/j.colsurfb.2013.02.033 23537829

[23] Rajendran, R. & Mani, A. Photocatalytic, antibacterial and anticancer activity of silver-doped zinc oxide nanoparticles. *J. Saudi Chem. Soc.* **24**(12), 1010–1024 (2020). 10.1016/j.jscs.2020.10.008

[24] Alijani, H. Q., Pourseyedi, S., Mahani, M. T. & Khatami, M. Green synthesis of zinc sulfide (ZnS) nanoparticles using stevia rebaudianaBertoni and evaluation of its cytotoxic properties. *J. Mol. Struct.* **1175**, 214–218 (2019). 10.1016/j.molstruc.2018.07.103

[25] Morshedtalab, Z. *et al.* Antibacterial assessment of zinc sulfide nanoparticles against *Streptococcus pyogenes* and *Acinetobacterbaumannii*. *Curr. Top. Med. Chem.* **20**(11), 1042–1055 (2020). 32250224 10.2174/1381612826666200406095246

[26] Tran, T. A., Krishnamoorthy, K., Cho, S. K. & Kim, S. J. Inhibitory effect of zinc sulfide nanoparticles towards breast cancer stem cell migration and invasion. *J. Biomed. Nanotechnol.* **12**(2), 329–336 (2016). 27305766 10.1166/jbn.2016.2187

[27] Govindasamy, G. A., Mydin, S. M. N. & R. B., Gadaime, N. K. R., & Sreekantan, S.,. Phytochemicals, biodegradation, cytocompatibility and wound healing profiles of chitosan film embedded green synthesized antibacterial ZnO/CuO nanocomposite. *J. Polym. Environ.* **31**(10), 4393–4409 (2023). 10.1007/s10924-023-02902-1

[28] Govindasamy, G. A., Mydin, R. B. S., Effendy, W. N. F. W. E. & Sreekantan, S. Novel dual-ionic ZnO/CuO embedded in porous chitosan biopolymer for wound dressing application: Physicochemical, bactericidal, cytocompatibility and wound healing profiles. *Mater. Today Commun.* **33**, 104545 (2022). 10.1016/j.mtcomm.2022.104545

[29] Govindasamy, G. A., Mydin, R. B. S., Sreekantan, S. & Harun, N. H. Compositions and antimicrobial properties of binary ZnO–CuO nanocomposites encapsulated calcium and carbon from *Calotropis**gigantea* targeted for skin pathogens. *Sci. Rep.* **11**(1), 99 (2021). 33420110 10.1038/s41598-020-79547-w PMC7794424

[30] Govindasamy, G. A., Mydin, R. B. S., Sreekantan, S. & Harun, N. H. Effect of calcination temperature on physicochemical and antimicrobial properties of green synthesised ZnO/C/Ca nanocomposites using *Calotropis**gigantea* leaves. *Adv. Natural Sci. Nanosci. Nanotechnol.* **12**(1), 015013 (2021). 10.1088/2043-6262/abe8da

[31] Govindasamy, G. A., Mydin, S. M. N. & R. B., Harun, N. H., Effendy, W. N. F. W. E., & Sreekantan, S.,. Giant milkweed plant-based copper oxide nanoparticles for wound dressing application: Physicochemical, bactericidal and cytocompatibility profiles. *Chem. Pap.* **77**(2), 1181–1200 (2023). 10.1007/s11696-022-02513-5

[32] Munir, T. *et al.* Impact of Ni dopant on optical and magnetic properties of ZnO nanoparticles for biomedical applications. *J. Ovonic Res.* **16**(3), 165–171 (2020). 10.15251/JOR.2020.163.165

[33] Munir, T., Latif, M., Mahmood, A., Malik, A. & Shafiq, F. Influence of IP-injected ZnO-nanoparticles in *Catla**catla* fish: Hematological and serological profile. *Naunyn-Schmiedeberg’s Arch. Pharmacol.* **393**, 2453–2461 (2020). 32725284 10.1007/s00210-020-01955-6

[34] Ravi, A. *et al.* Tunable band gap energy of l-Cysteine-assisted formation of Mn-doped ZnS interconnected nanoparticles for electro-optic applications. *Opt. Mater.* **150**, 115293 (2024). 10.1016/j.optmat.2024.115293

[35] Devi, B. L., Kompa, A., Rao, K. M. & Ramananda, D. Spectroscopic analysis of Cu doped and starch capped ZnS nanocrystals synthesized by microwave irradiation. *Ceram. Int.* **50**(6), 8890–8895 (2024). 10.1016/j.ceramint.2023.12.204

[36] Kumari, P., Misra, K. P., Samanta, S. & Chattopadhyay, S. Photoluminescence, morphology and band gap in Europium-doped ZnS nanoparticles. *Mater. Technol.* **39**(1), 2286822 (2024). 10.1080/10667857.2023.2286822

[37] Rajivgandhi, G. *et al.* Preparation of antibacterial Zn and Ni substituted cobalt ferrite nanoparticles for efficient biofilm eradication. *Anal. Biochem.* **653**, 114787 (2022). 35709929 10.1016/j.ab.2022.114787

[38] Rajivgandhi, G. N. *et al.* Substantial effect of Cr doping on the antimicrobial activity of ZnO nanoparticles prepared by ultrasonication process. *Mater. Sci. Eng. B* **263**, 114817 (2021). 10.1016/j.mseb.2020.114817

[39] Gnanamozhi, P. *et al.* Enhanced antibacterial and photocatalytic degradation of reactive red 120 using lead substituted ZnO nanoparticles prepared by ultrasonic-assisted co-precipitation method. *Ceram. Int.* **46**(11), 19593–19599 (2020). 10.1016/j.ceramint.2020.05.020

[40] Maruthupandy, M. *et al.* Graphene-zinc oxide nanocomposites (G–ZnO NCs): Synthesis, characterization and their photocatalytic degradation of dye molecules. *Mater. Sci. Eng. B* **254**, 114516 (2020). 10.1016/j.mseb.2020.114516

[41] Rajivgandhi, G., Maruthupandy, M., Muneeswaran, T., Anand, M. & Manoharan, N. Antibiofilm activity of zinc oxide nanosheets (ZnO NSs) using *Nocardiopsis* sp. GRG1 (KT235640) against MDR strains of gram negative *Proteus mirabilis* and *Escherichia coli*. *Process Biochem.* **67**, 8–18 (2018). 10.1016/j.procbio.2018.01.015

[42] Mahmood, A., Munir, T., Rasul, A., Ghfar, A. A. & Mumtaz, S. Polyethylene glycol and chitosan functionalized manganese oxide nanoparticles for antimicrobial and anticancer activities. *J. Coll. Interface Sci.* **648**, 907–915 (2023). 10.1016/j.jcis.2023.06.029 37329602

[43] Munir, T., Mahmood, A., Rasul, A., Imran, M. & Fakhar-e-Alam, M. Biocompatible polymer functionalized magnetic nanoparticles for antimicrobial and anticancer activities. *Mater. Chem. Phys.* **301**, 127677 (2023). 10.1016/j.matchemphys.2023.127677

[44] Alwany, A. B. *et al.* Structural, optical and radiation shielding properties of ZnS nanoparticles QDs. *Optik* **260**, 169124 (2022). 10.1016/j.ijleo.2022.169124

[45] Kripal, R. *et al.* Photoluminescence and photoconductivity of ZnS: Mn2+ nanoparticles synthesized via co-precipitation method. *Spectrochim. Acta Part A Mol. Biomol. Spectrosc.* **76**(5), 523–530 (2010). 10.1016/j.saa.2010.04.018 20452818

[46] Juine, R. N., Das, A. & Amirthapandian, S. Concentration controlled QDs ZnS synthesis without capping agent and its optical properties. *Mater. Lett.* **128**, 160–162 (2014). 10.1016/j.matlet.2014.03.104

[47] Sun, J. Q., Shen, X. P., Chen, K. M., Liu, Q. & Liu, W. Low-temperature synthesis of hexagonal ZnS nanoparticles by a facile microwave-assisted single-source method. *Solid State Commun.* **147**(11–12), 501–504 (2008). 10.1016/j.ssc.2008.06.041

[48] Aziz, A. *et al.* Chitosan-zinc sulfide nanoparticles, characterization and their photocatalytic degradation efficiency for azo dyes. *Int. J. Biol. Macromol.* **153**, 502–512 (2020). 32126200 10.1016/j.ijbiomac.2020.02.310

[49] Dawngliana, K. M. S., Ralte, L. & Rai, S. Effect of ZnS nanoparticles on the optical properties of Sm^3+^ ions in silicate matrix. *J. Non Cryst. Solids* **632**, 122871 (2024). 10.1016/j.jnoncrysol.2024.122871

[50] Kaur, J., Sharma, M. & Pandey, O. P. Structural and optical studies of undoped and copper doped zinc sulphide nanoparticles for photocatalytic application. *Superlattices Microstruct.* **77**, 35–53 (2015). 10.1016/j.spmi.2014.10.032

[51] Jothibas, M. *et al.* Synthesis and enhanced photocatalytic property of Ni doped ZnS nanoparticles. *Sol. Energy* **159**, 434–443 (2018). 10.1016/j.solener.2017.10.055

[52] Selvaraj, V. *et al.* Enhanced photodegradation of methylene blue from aqueous solution using Al-doped ZnS nanoparticles. *Environ. Sci. Pollut. Res.* **29**(48), 73528–73541 (2022). 10.1007/s11356-022-20634-y 35622286

[53] Alwany, A. B. *et al.* Effect of lead doping on the structural, optical, and radiation shielding parameters of chemically synthesized ZnS nanoparticles. *J. Mater. Sci. Mater. Electron.* **34**(3), 233 (2023). 10.1007/s10854-022-09647-y

[54] Dash, S. K., Ghosh, T., Roy, S., Chattopadhyay, S. & Das, D. Zinc sulfide nanoparticles selectively induce cytotoxic and genotoxic effects on leukemic cells: Involvement of reactive oxygen species and tumor necrosis factor alpha. *J. Appl. Toxicol.* **34**(11), 1130–1144 (2014). 24477783 10.1002/jat.2976

[55] Munir, T. *et al.* Structural, morphological and optical properties at various concentration of Ag doped SiO_2_–NPs via sol gel method for antibacterial and anticancer activities. *Surf. Interfaces* **38**, 102759 (2023). 10.1016/j.surfin.2023.102759

[56] Wei, X., Wang, W. & Chen, K. Preparation and characterization of ZnS: Tb, Gd and ZnS: Er, Yb, Gd nanoparticles for bimodal magnetic-fluorescent imaging. *Dalton Trans.* **42**(5), 1752–1759 (2013). 23160019 10.1039/C2DT31783D

[57] Dhupar, A. *et al.* In-doped ZnS nanoparticles: Structural, morphological, optical and antibacterial properties. *Appl. Phys. A* **127**, 1–11 (2021). 10.1007/s00339-021-04425-9

[58] Muthuchamy, M. *et al.* Biologically synthesized copper and zinc oxide nanoparticles for important biomolecules detection and antimicrobial applications. *Mater. Today Commun.* **22**, 100766 (2020). 10.1016/j.mtcomm.2019.100766

[59] Rajivgandhi, G. *et al.* Biologically synthesized copper oxide nanoparticles enhanced intracellular damage in ciprofloxacin resistant ESBL producing bacteria. *Microb. Pathog.* **127**, 267–276 (2019). 30550842 10.1016/j.micpath.2018.12.017

