# Supplementary material for: Investigation of antibacterial and anticancer activities of copper, aluminum and nickel doped zinc sulfide nanoparticles
Source: Sci Rep. 2024 Aug 20;14:19304. doi: 10.1038/s41598-024-68631-0 (PMC11335939; doi:10.1038/s41598-024-68631-0)
Supplement: Supplementary file 1 — Supplementary Figures. [file 41598_2024_68631_MOESM1_ESM.docx]

Supplementary figure 1: SEM micrographs

| 1. 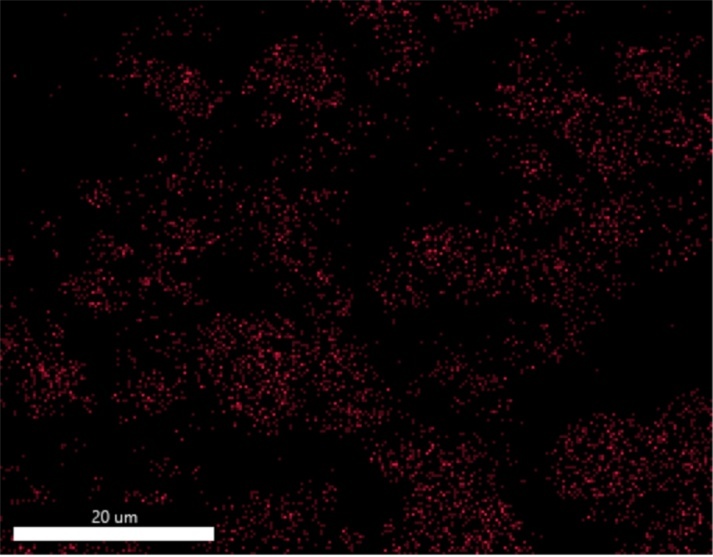**Zinc** | 1. 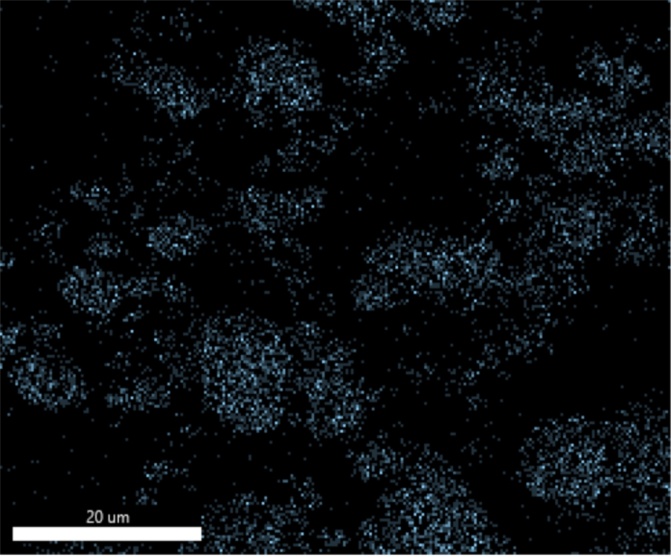**Sulfur** |
| --- | --- |
| 1. 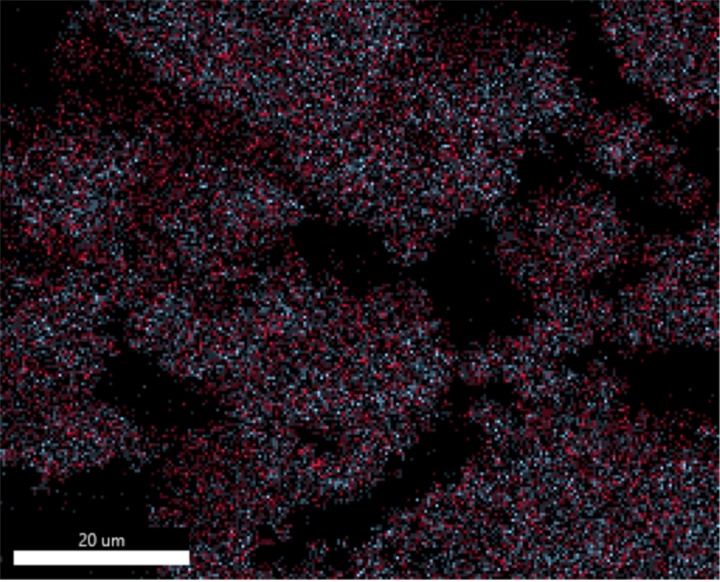**Cu doped ZnS NPs** | 1. 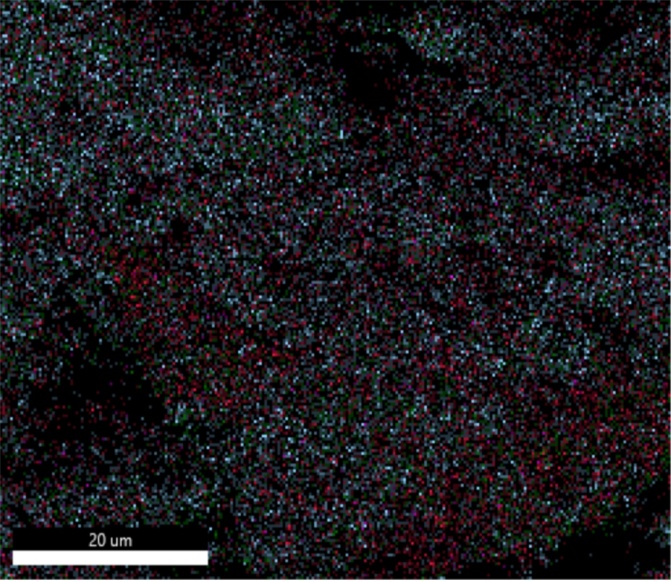Al doped ZnS NPs |
| 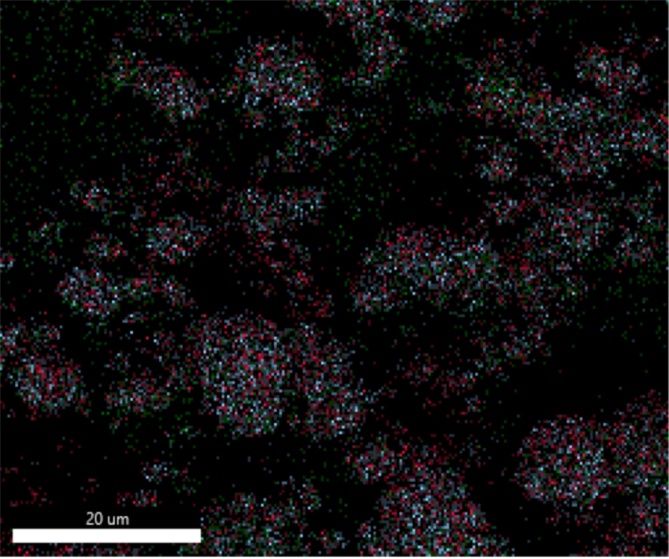 **(E) Ni doped ZnS NPs** | |

**Supplementary figure 2: Inhibition Zone of *E.coli* and *B.cereus***

| 1. 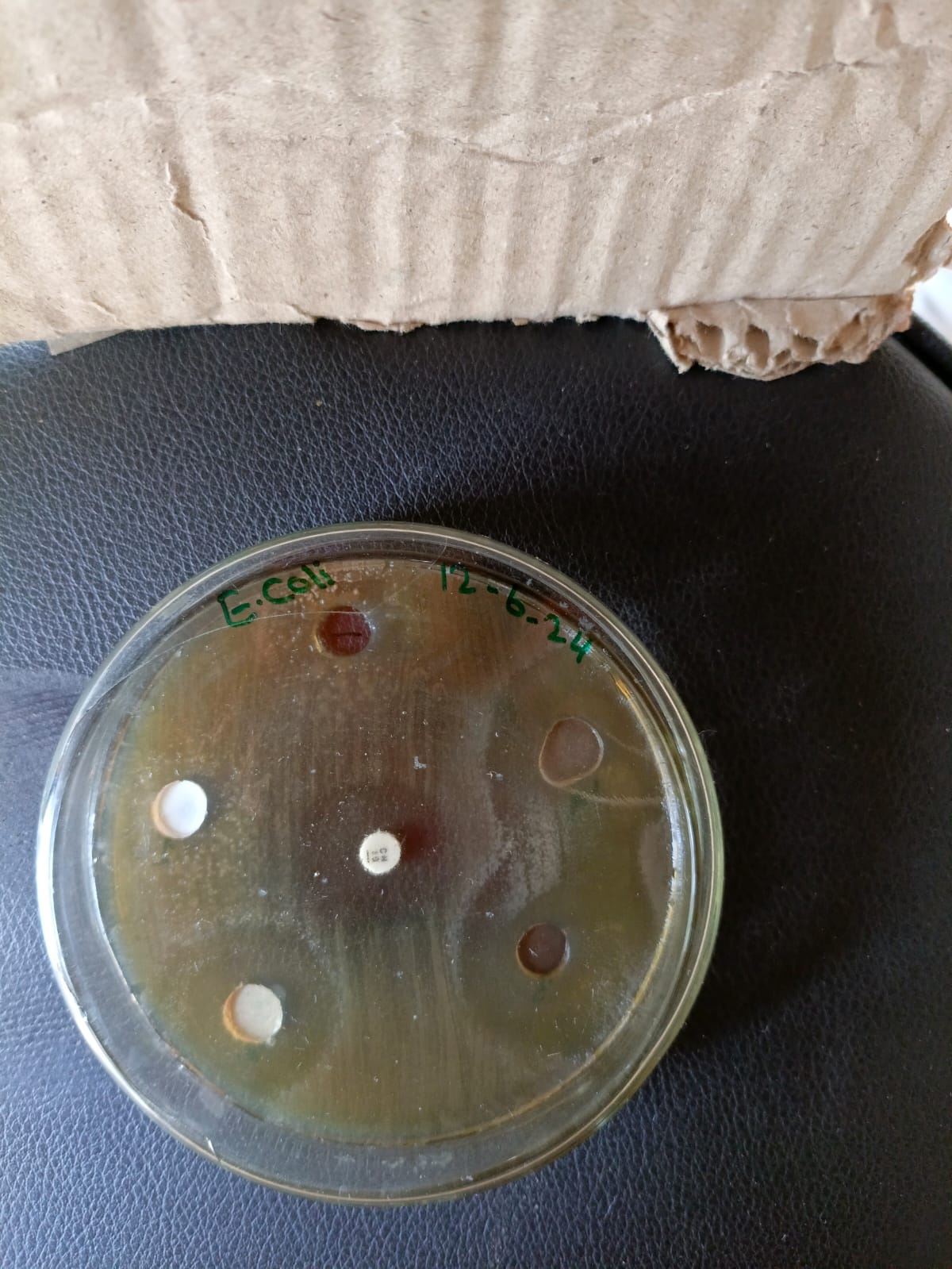 *E.coli* (Front) 20 mg | 1. *E.coli* (Back) 20 mg 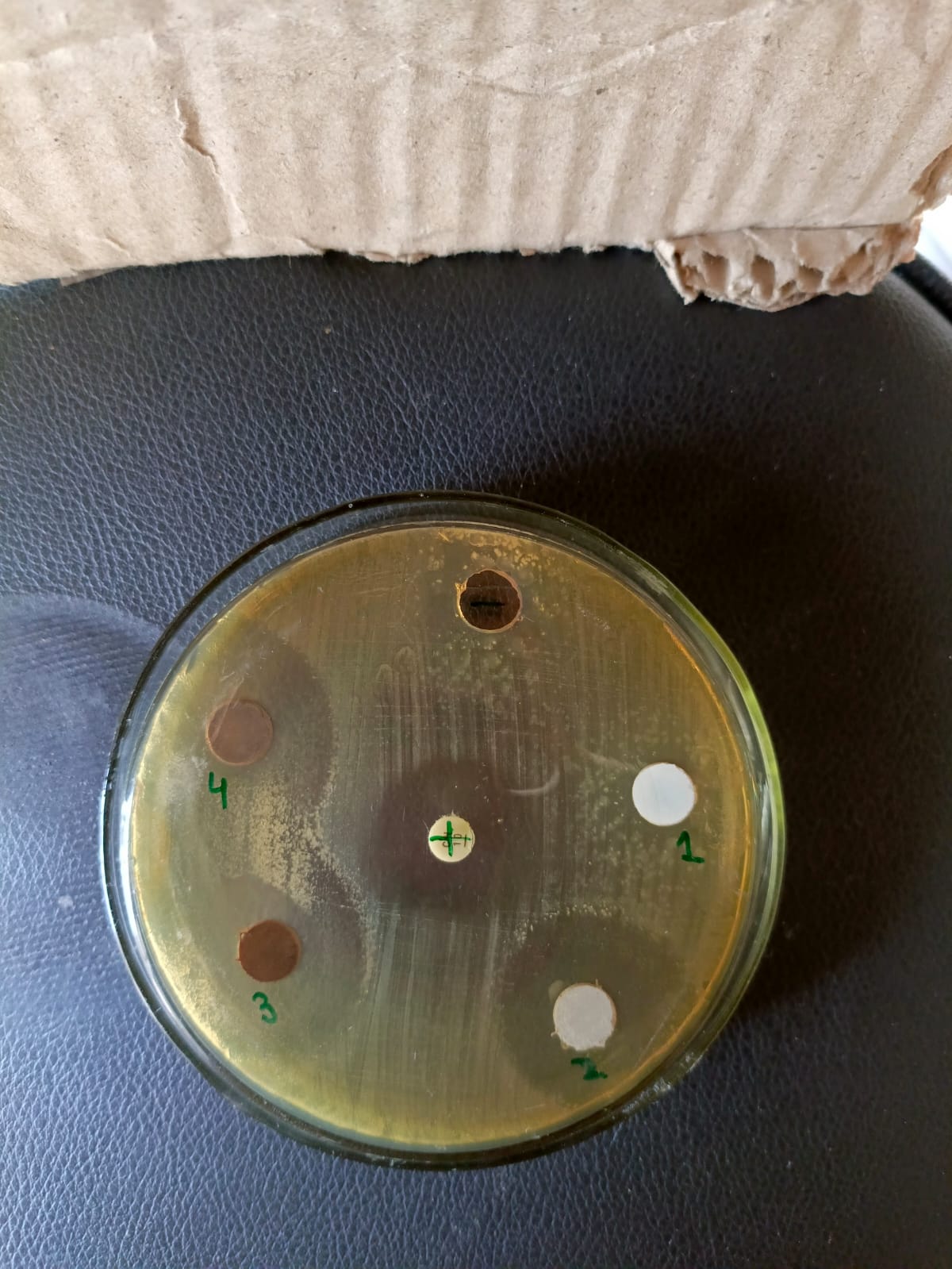 |
| --- | --- |
| 1. 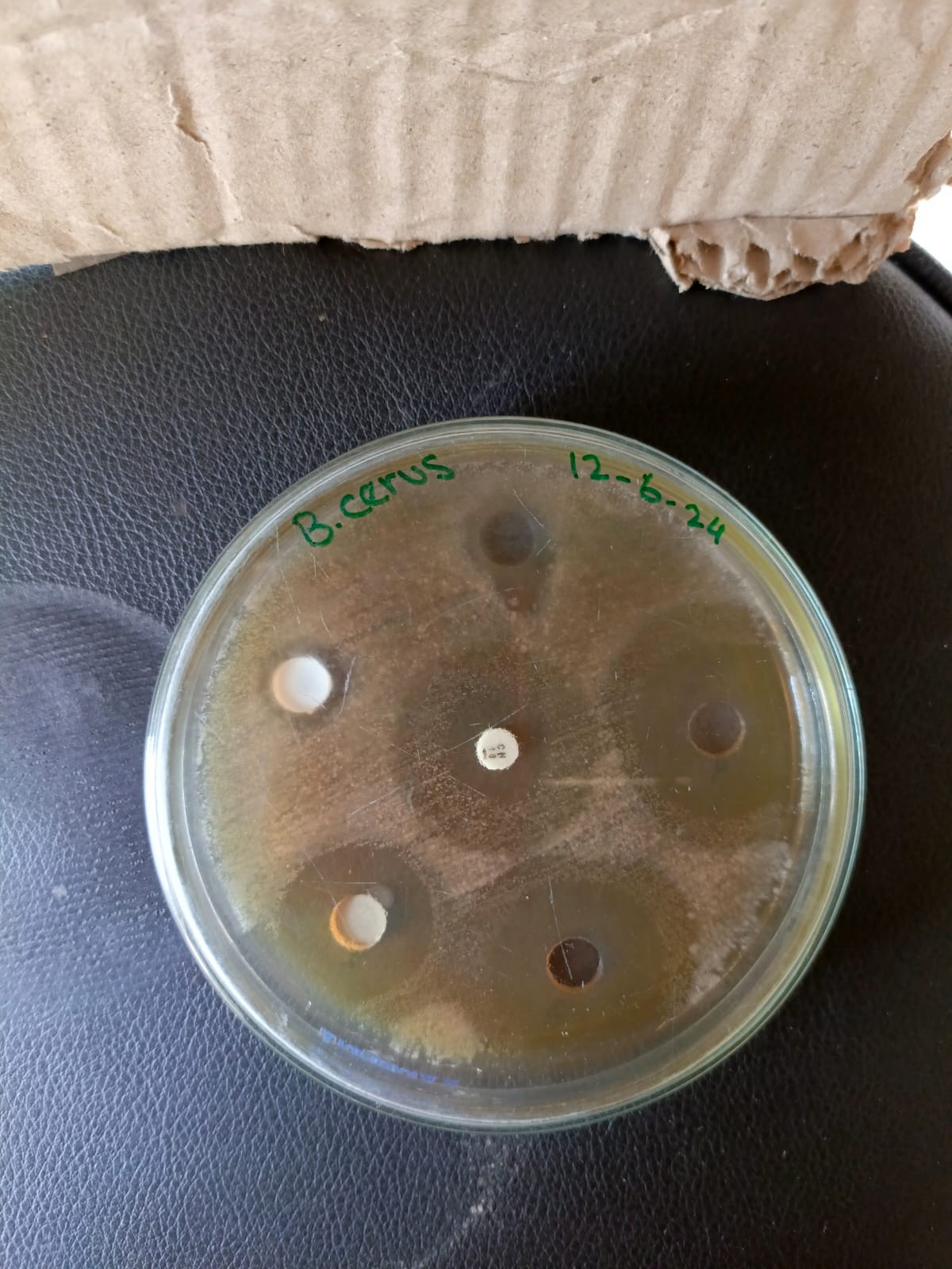 *B.cerus* (Front) 20 mg | 1. *B.cerus* (Back) 20 mg 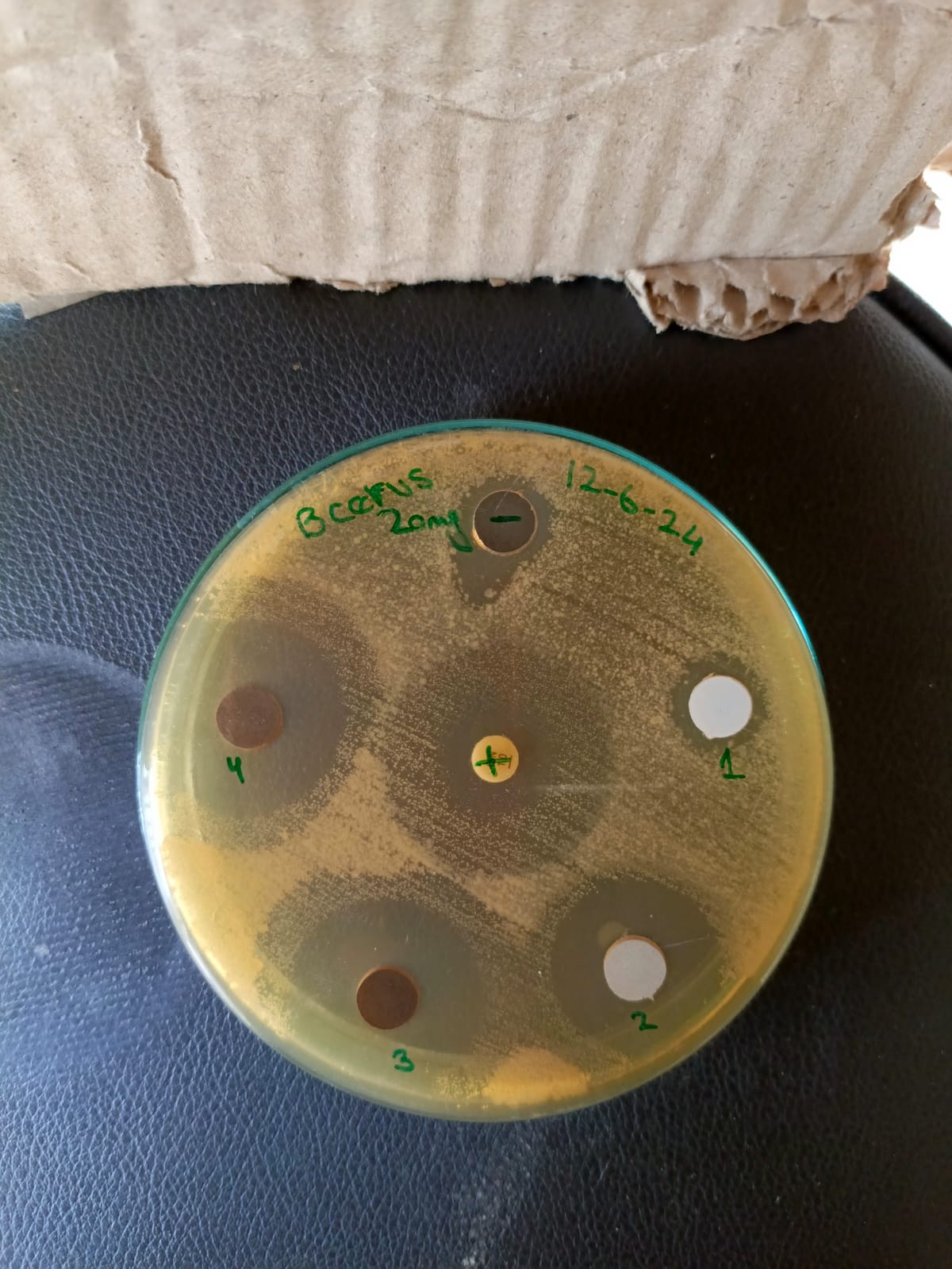 |
